# Circulating dendritic cell levels identify high-risk stage II-III melanoma patients: a potential role as additional prognostic marker

**DOI:** 10.1186/1479-5876-13-S1-P14

**Published:** 2015-01-15

**Authors:** Stefania Stucci, Marco Tucci, Anna Passarelli, Francesco Mannavola, Claudia Felici, Giuseppe Giudice, Franco Silvestris

**Affiliations:** 1 Medical Oncology Unit - DIMO, University of Bari ‘Aldo Moro’, Italy; 2 Plastic Surgery Unit– DETO, University of Bari ‘Aldo Moro’, Italy

## Background

Melanoma is an immunogenic cancer that overcomes the immune surveillance through the production of tolerogenic cytokines and growth factors within microenvironment. Melanoma-derived dendritic cells (DCs) show altered maturation, cross-priming and antigenic presentation and their major defect concerns the activation of the STAT pathway. The prognostic criteria to define melanoma at high-risk of relapse/recurrence include the Breslow depth, the Clark level and number of mitosis. Sentinel lymph node (SLN) characterization is a prognostic factor in melanoma, although false negative occurs in approximately 5% of patients. We investigated the potential prognostic role of DC number variation in relation to clinical stage, and suggest their role as early predictor of high risk melanomas.

## Methods

84 patients (group A; stage I-IV) with a previously definite diagnosis of melanoma as well as 18 (group B) with highly suspected cutaneous lesion were enrolled into the study. Peripheral blood was collected at study entry in patients of group A, whereas cells from group B were collected before and after the primary tumor exeresis and then before/after any surgical procedure including SLN, lymphadenectomy or metastasis removal. Both myeloid (m) and plasmacytoid (p) DCs were investigated by flow-cytometry using the anti-Lin, -CD11c, -BDCA-1, -CD123, and -BDCA-2 MoAbs. The percentage number of both mDCs and pDCs was correlated to clinical stage as well as to indipendent prognostic factors as histological features including Breslow, Clark level, presence/absence of tumor-infiltrating lymphocytes, and BRAF V600^E/K^ mutations.

## Results

The percentage of both mDCs and pDCs from group A were similar in stage I-III and dramatically reduced in those at stage IV. A. Lack of correlation was also demonstrated with clinical features and prognostic factors. By contrast, data from group B showed: an increase of mDCs and pDCs (p<0.05) after tumor removal as well as in those with negative SLN whereas both subsets resulted unchanged in patients with positive SLN. A weak trend to DC increase occurred in patients undergoing negative lymphoadenectomy. Number of mDCs and pDCs from group B was correlated with histological features and unrelated to mutational status.

## Conclusions

These findings suggest the critical pathogenetic role of DC in melanoma and their measurement may be thus proposed as additional prognostic factor to limit the risk of underscoring the melanoma stage of patients at high risk of relapse/recurrence.

